# Substrate Specificity and Biochemical Characteristics of an Engineered Mammalian Chondroitinase ABC

**DOI:** 10.1021/acsomega.0c06262

**Published:** 2021-04-19

**Authors:** Philippa M. Warren, James W. Fawcett, Jessica C. F. Kwok

**Affiliations:** † Department of Clinical Neurosciences, John van Geest Centre for Brain Repair, University of Cambridge, Cambridge CB2 0PY, U.K.; ‡ Wolfson Centre for Age Related Diseases, Institute of Psychiatry, Psychology and Neuroscience, King’s College London, Guy’s Campus, London Bridge, London SE1 1UL, U.K.; § Department of Physiology, Development and Neuroscience, University of Cambridge, Cambridge CB2 0PY, U.K.; ∥ Centre for Reconstructive Neuroscience, Institute of Experimental Medicine, Czech Academy of Sciences, Videnska 1083, 14220 Prague 4, Czech Republic; ⊥ School of Biomedical Sciences, Faculty of Biological Sciences, University of Leeds, Leeds LS2 9JT, U.K.

## Abstract

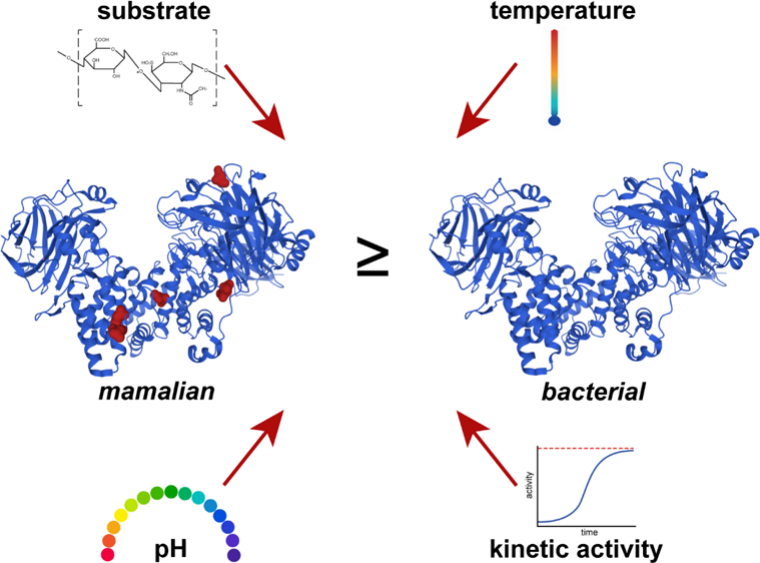

Chondroitin sulfate proteoglycans inhibit regeneration, neuroprotection, and plasticity following spinal cord injury. The development of a second-generation chondroitinase ABC enzyme, capable of being secreted from mammalian cells (mChABC), has facilitated the functional recovery of animals following severe spinal trauma. The genetically modified enzyme has been shown to efficiently break down the inhibitory extracellular matrix surrounding cells at the site of injury, while facilitating cellular integration and axonal growth. However, the activity profile of the enzyme in relation to the original bacterial chondroitinase (bChABC) has not been determined. Here, we characterize the activity profile of mChABC and compare it to bChABC, both enzymes having been maintained under physiologically relevant conditions for the duration of the experiment. We show that this genetically modified enzyme can be secreted reliably and robustly in high yields from a mammalian cell line. The modifications made to the cDNA of the enzyme have not altered the functional activity of mChABC compared to bChABC, ensuring that it has optimal activity on chondroitin sulfate-A, with an optimal pH at 8.0 and temperature at 37 °C. However, mChABC shows superior thermostability compared to bChABC, ensuring that the recombinant enzyme operates with enhanced activity over a variety of physiologically relevant substrates and temperatures compared to the widely used bacterial alternative without substantially altering its kinetic output. The determination that mChABC can function with greater robustness under physiological conditions than bChABC is an important step in the further development of this auspicious treatment strategy toward a clinical application.

## Introduction

Promoting functional and anatomical regeneration following spinal cord injury (SCI) is therapeutically challenging. A major obstacle to any form of recovery is the inhibitory environment that develops around the lesion site, dominated by the presence of chondroitin sulfate proteoglycans.^1−3^ The bacterial enzyme chondroitinase ABC (bChABC) acts by cleaving the chondroitin sulfate-glycosaminoglycan (CS-GAG) chains into their component disaccharides and subsequently removing what is considered to be the major inhibitory component of these macromolecules.^4−7^ Treatment using this enzyme (originally isolated from*Proteus vulgaris*) has been successful at promoting plasticity and regeneration *in vivo* and *in vitro* following experimental SCI in a number of different models.^8−17^ However, several factors limit the clinical use of the enzyme *in vivo*. For example, bChABC is temperature-sensitive, losing most activity within 3 to 10 days at 37 °C.^18,19^ For the more severe SCIs, a single bolus injection of the enzyme is not sufficient to yield functional recovery,^20−22^ and a more invasive treatment or repeated administration is required, such as intrathecal infusion or secretion of the enzyme from an implanted biomaterial.^23−26^

Gene therapy has often been used to facilitate the experimental sustained delivery of therapeutic molecules through *in vivo* or *ex vivo* strategies. Cafferty et al.^27^ demonstrated these techniques *in vivo* through the transgenic expression of bChABC cDNA in mouse reactive astrocytes under the GFAP promoter, facilitating the decrease of CS-GAGs at the site of SCI. However, the *N*-glycosylation system in eukaryotes has limited the secretion of the bacterial enzyme in mammalian cells.^27^ A recombinant form of bChABC capable of being transduced and secreted in an active form from mammalian cells (mChABC) has been developed through the mutagenesis of key *N*-glycosylation sites on the molecule.^28,29^ Recently, it has been shown that targeting this mChABC construct to axons can promote neurite extension *in vitro*.^30^ Using a lenti-viral version of mChABC, we have shown that enzyme-transduced Schwann cells are able to migrate and intermingle within astrocytic boundaries, facilitating neurite outgrowth *in vitro* and *in vivo*.^29^ Following acute lenti-viral transduction into the injured spinal cord, mChABC was shown to be secreted, active, and able to produce functional sprouting and motor recovery up to 10 weeks following trauma *in vivo*.^31,32^ In combination with the development of viral technology, mChABC has recently been expressed successfully in an immune-evasive regulatable adeno-associated viral vector under doxycycline induction.^32^ This provides an important pathway for the potential translation of ChABC in gene therapy. However, neither the biochemical characteristics of mChABC under physiologically relevant conditions nor the relationship it has with those of the commercial bChABC has been determined. This is despite *in vitro* and *in vivo* evidence that the recombinant enzyme may be more effective than bChABC.^29^

It is important to assess the characteristics of mChABC to determine if it operates effectively in conditions likely to be experienced within the human body. These data may impact the development of the mChABC construct for clinical treatments. Furthermore, it is important to determine if the modifications made to the recombinant enzyme have caused alterations in its potential activity and effectiveness. Here, we use transfection of a plasmid-mChABC to show the ease of enzyme expression in, and secretion from, mammalian cells. We report the optimal biochemical conditions and enzyme kinetics for the secreted mChABC enzyme under physiologically relevant conditions and demonstrate that the recombinant enzyme has superior functionality than bChABC due to increased thermostability.

## Results and Discussion

### Transfection of HEK293 Cells Yields High Quantities of Thermostable mChABC

**Figure 1:**
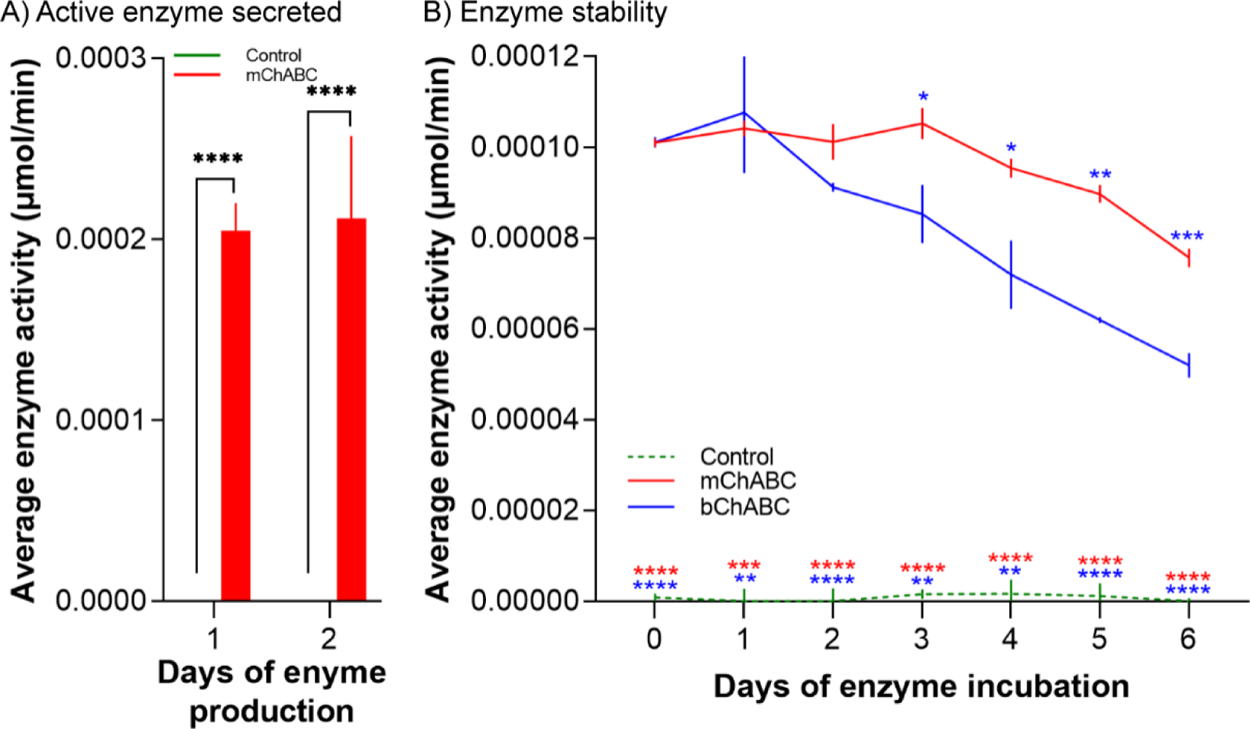
Amount and stability of active mChABC secreted by HEK293 cells. (A) Average amount of active mChABC secreted from HEK293 over a 24 h period for 2 consecutive days following transfection (*N* = 3 from independent cell batches). (B) Stability of 0.0001 μmol/min mChABC over a period of 6 days at 37 °C (*N* = 3 from independent cell batches). For both panels, data show means ± SD.

In order to assess the activity of mChABC, HEK293 cells were transfected with the plasmid mChABC (*p*-mChABC) construct through nucleofection (Supporting Information Figure S1A–C). The concentrated lysate was collected over 24 h following transfection and assessed for mChABC activity using the cetylpyridinium chloride (CPC) turbidity assay. A population of 2 × 10^6^ cells transfected with *p*-mChABC consistently produced a yield of 0.0002 μmol/min active enzyme as compared to control populations, which yielded no such activity (Figure 1A). The day of collection following transfection did not affect the amount of the active enzyme produced [F(1,4) = 0.0163, *p* = 0.905, two-way ANOVA with post-hoc Turkey], suggesting that the cells can regularly secrete similar amounts of the molecule over time. To assess the stability of the enzyme, 0.0001 μmol/min of mChABC and bChABC was incubated at 37 °C for 6 days with activity measured daily. The secreted mChABC was shown to have superior thermostability in culture medium compared to the commercially available bChABC, remaining at a plateau of activity for 5 rather than 2 days and exhibiting less loss of total activity over time [Figure 1B; F(12,36) = 19.2, *P* < 0.0001, two-way ANOVA post-hoc Turkey]. These data demonstrate that biologically active mChABC is expressed and secreted from transfected cells with high stability.

### Biochemical Characterization of mChABC Activity

Having established mChABC expression in HEK293 cells, the optimal biochemical conditions to achieve maximal activity of the mammalian enzyme were determined and assessed against the commercially available bChABC. These parameters included substrate specificity, pH, temperature, and thermostability. To ensure accuracy when comparing the mChABC and bChABC, the commercial enzyme was placed under the same conditions as the recombinant enzyme for the same length of time [Dulbecco’s modified Eagle’s medium (DMEM) with sodium pyruvate and ITS+, at 37 °C for 24 h, centrifuged, EDTA-free protease inhibitor cocktail added, concentrated, and quantity of the active enzyme assessed through the CPC turbidity assay].

#### Optimal Substrate

Concentrations of 0.5 μg/μL active mChABC and bChABC were determined using the CPC turbidity assay and used to assess the activity of the ChABCs on CS-A, -C, DS, and HA (Table 1; Supporting Information Figure S2). These data showed that both the specific ChABC used [F(2,24) = 555,836, *P* < 0.0001, two-way ANOVA with post-hoc Bonferroni] and the substrate under consideration [F(3,24) = 205,278, *P* < 0.0001, two-way ANOVA with post-hoc Bonferroni] affected the degree of enzyme activity. Interestingly, both mChABC and bChABC exhibited minimal levels of activity on HA at pH 8.0, showing no difference from control (*P* = ns; Table 1). However, mChABC showed activity on CS-A, CS-C, and DS, which was ∼25% higher than that achieved with the bChABC (Table 1; *P* < 0.0001 in all comparisons). Both mChABC and bChABC showed an ∼25% decrease in activity using CS-C and ∼50% reduction with DS substrates as compared to CS-A (Table 1),^6,33,34^ clearly establishing the latter as the optimal substrate. These data confirm that the modifications to mChABC have not altered the enzyme substrate binding properties, explaining why it has effects similar to the commercial enzyme *in vivo*. However, mChABC shows greater activity than the bacterial enzyme under the same conditions.

**Table 1:** Activity of Commercial bChABC and mChABC Acting on GAG Substrates^a^

substrate	average MW (kDa)	control (μmol min^–1^ mg^–1^)	mChABC (μmol min^–1^ mg^–1^)	bChABC (μmol min^–1^ mg^–1^)
CS-A from bovine trachea	12	0.0558 ± 0.000185	4.69 ± 0.00614	3.35 ± 0.00596
DS from porcine intestinal mucosa	16	0.0264 ± 0.000573	2.57 ± 0.0086	2.23 ± 0.00167
CS-C from shark cartilage	35	0.0147 ± 0.00019	3.57 ± 0.00436	2.95 ± 0.00334
Hyaluronan	215	0.0117 ± 0.000173	0.444 ± 0.00131	0.429 ± 0.0014

a Enzyme activity measured as U/mg of protein (μmol min^–1^ mg^–1^). Values show means ± SD and shown to three significant figures. The experiment was conducted at pH 8 and 37 °C (*N* = 3 for each condition from independent cell batches). Sigma bChABC was preincubated at 37 °C for 48 h prior to assessment. MW = molecular weight.

#### Optimal pH

**Figure 2:**
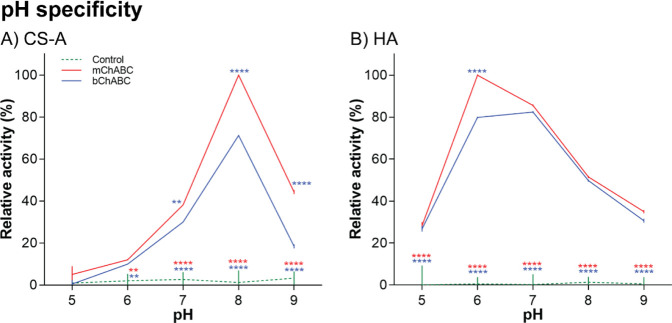
pH specificity of mChABC compared to bChABC. Effect of pH on enzyme activity when incubated with (A) CS-A and (B) hyaluronic acid (HA). For both panels, data show means ± SD and *N* = 3 (from independent cell batches).

The pH range at which the enzymes maximally operate was evaluated using both CS-A and HA as substrates and the relative activity reported as compared to the maximum obtained. mChABC and bChABC showed activity greater than the control in all experimental conditions (Figure 2). Both enzymes exhibited a relatively narrow pH-activity profile of ±0.5 pH around the optimum pH 8.0 on CS-A [Figure 2A; F(4,30) = 446.7, *P* < 0.0001, two-way ANOVA with post-hoc Turkey]. However, mChABC showed ∼25% greater activity compared to the bChABC over this pH range [F(2,30) = 591.3, *P* < 0.0001, two-way ANOVA with post-hoc Turkey]. These data again suggest that the engineered mChABC enzyme functions more effectively than the commercial alternative. The optimum activity on HA for both ChABCs occurred at pH 6.0 [Figure 2B; F(4,30) = 345.2, *P* < 0.0001, two-way ANOVA with post-hoc Turkey].^4,6,33−35^ Both mChABC and bChABC were relatively active over a wider pH range than displayed on CS-A (pH 5–8), although enzyme activity was substantially reduced (Figure 2B; Table 1). Interestingly, when outside the optimal pH range (and thus under different conditions from those in Table 1), mChABC showed increased activity on HA compared to bChABC [*P* < 0.0001; Figure 2B; F(2,30) = 1678, *P* < 0.0001, two-way ANOVA with post-hoc Turkey]. These data show that mChABC may function better under the physiological conditions of the mammalian body than bChABC. All subsequent assays were performed at the optimized pH 8.0 on CS-A.

#### Optimal Temperature

**Figure 3:**
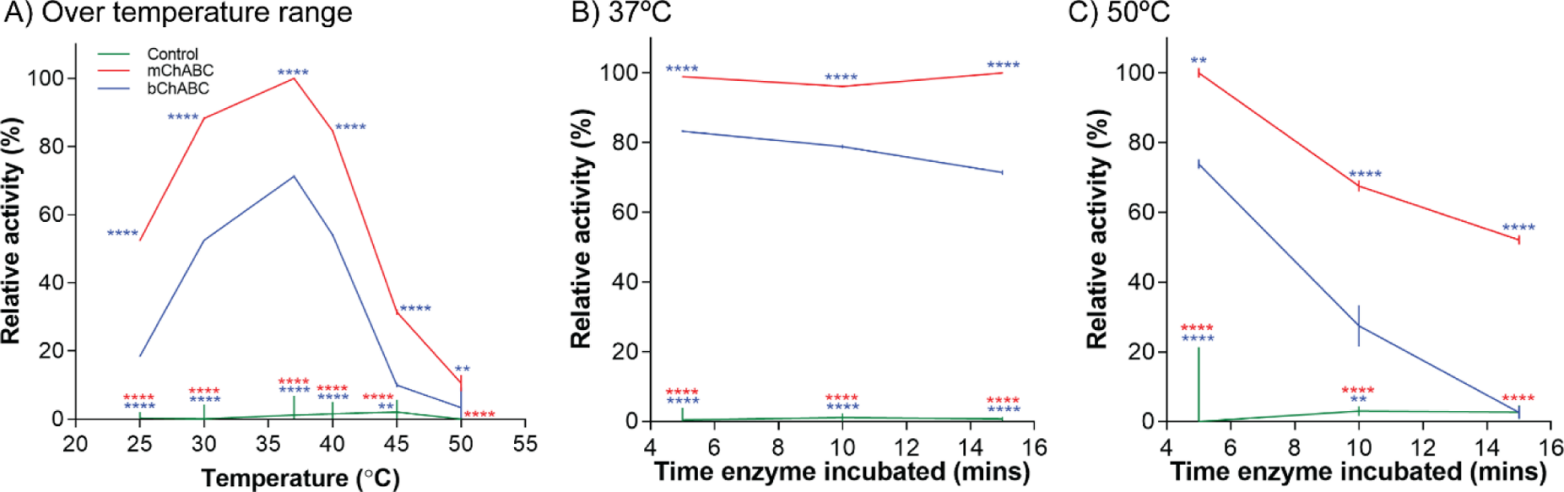
Temperature specificity of mChABC compared to bChABC. Effect of temperature on enzyme activity using CS-A at pH 8 over (A) a temperature range, and preincubation of the enzyme at (B) 37 °C and (C) 50 °C to assess thermostability. For all panels, data show means ± SD and *N* = 3 (from independent cell batches).

We showed that both the specific ChABC used [F(2,36) = 2468, *P* < 0.0001, two-way ANOVA with post-hoc Turkey] and the temperature at which the assay is conducted [F(5,36) = 596.1, *P* < 0.0001, two-way ANOVA with post-hoc Turkey] affected enzyme activity (Figure 3). Both mChABC and bChABC showed optimal activity at the physiological temperature of 37 °C (Figure 3A). Furthermore, the activity profile of the two enzymes over the temperature range was similar with an ∼40% increase in relative activity over 25–37 °C, and a rapid decline in activity as temperatures increased. Indeed, the ChABC enzymes showed an ∼50% loss in relative activity in temperatures exceeding 40 °C. However, consistent with our previous data, mChABC showed an ∼25% greater relative activity than bChABC across this temperature range (Figure 3A; minimum *P* < 0.01). This is again indicative of the greater activity and stability of the recombinant enzyme, suggesting that it may be more effective than the commercial alternative *in vivo*.

To determine if the rapid decline in mChABC and bChABC relative activity at higher temperatures was caused by irreversible denaturation, the enzymes were incubated for 5, 10, and 15 min at 37 or 50 °C and then transferred to 37 °C for data acquisition (Figure 3B,C). Interestingly, data show that mChABC was relatively stable at 37 °C, more so than bChABC, the latter showing a gradual decline in relative activity with continued incubation (Figure 3B; variation due to the enzyme used F(2,18) = 14,404, *P* < 0.0001; variation due to time F(2,18) = 17.02, *P* < 0.0001, two-way ANOVA with post-hoc Turkey). The trends in data are also shown at 50 °C [Figure 3C; variation due to the enzyme used F(2,18) = 203.1, *P* < 0.0001; variation due to time F(2,18) = 61.93, *P* < 0.0001, two-way ANOVA with post-hoc Turkey]. Under these conditions, the relative activity of mChABC falls by ∼25% for every 5 min of incubation. The decline in bChABC activity is far more rapid with a ∼45% decrease for every 5 min of incubation, occurring under minimal baseline conditions following 15 min incubation (*P* = 0.9998; Figure 3C). These data suggest that denaturation is a factor in ChABC activity at temperatures above 40 °C and that mChABC is more thermostable than the commercial enzyme at all physiologically relevant temperatures assessed.

### mChABC Kinetic Activity

Kinetic parameters were determined for the engineered mChABC enzyme and compared to the commercial bChABC (Table 2). The kinetic parameters used to characterize enzyme activity each showed differences due to the enzyme used. This included the concentration of the substrate leading to half-maximal velocity [*K*_m_; F(2,21) = 471.5, *P* < 0.0001, one-way ANOVA with post-hoc Turkey] the number of substrate molecules the enzyme converts to product per unit time [*k*_cat_; F(2,21) = 13,428, *P* < 0.0001, one-way ANOVA with post-hoc Turkey]; and the catalytic efficiency [*k*_cat_/*K*_m_; F(2,21) = 135.1, *P* < 0.0001, one-way ANOVA with post-hoc Turkey]. mChABC showed a decrease in CS-A binding affinity (*K*_m_) compared to bChABC (Table 2). However, the engineered enzyme showed a large increase (*P* < 0.0001) in values describing the catalytic center of activity (*k*_cat_). This means that mChABC may bind with slightly less frequency to the substrate than bChABC. However, following binding, mChABC catabolizes the molecule with greater frequency than bChABC. For this reason, there is no significant difference between the catalytic efficiency (*k*_cat_/*K*_m_) of mChABC and bChABC (*P* = 0.216; Table 2), suggesting that the reaction rate of the enzymes is similar under optimal conditions.

**Table 2:** Kinetic Analysis of mChABC and Commercial bChABC^a^

enzyme	*K*_m_ (μM)	*k*_cat_ (min^–1^)	*k*_cat_/*K*_m_ (μM^–1^ min^–1^)
no enzyme control	–0.0384 ± 0.147	0.163 ± 0.0372	–4.24
mChABC	0.527 ± 0.0523	46.1 ± 0.874	84.5
bChABC (Sigma)	0.373 ± 0.0318	37.1 ± 0.544	99.4

a Values show means ± SD. *K*_m_ and *k*_cat_ are shown to three significant figures and *k*_cat_/*K*_m_ to 1 decimal place. *N* = 8 for each condition (from independent cell batches).

We have established the biochemical characteristics of a mammalian-compatible ChABC in direct comparison with the commercial bacterial alternative. We show that delivery of the recombinant mChABC into mChABC leads to high expression and secretion of the active enzyme *in vitro*. The resulting mChABC produced was shown to have superior biological activity on a variety of physiological substrates, while maintaining the enzymes optimal temperature and pH range. While the genetic alterations made to mChABC were shown to modify individual components of the enzyme catalytic reaction, ultimately its kinetic capacity is no different from the commercially available bChABC. However, we demonstrate that due to superior thermostability, mChABC has significantly greater activity then bChABC under physiological conditions, supporting its use *in vivo* and continued development as a clinical treatment.

The large-scale expression and secretion of active mChABC from cell lines has not previously been shown. We demonstrate that mChABC is secreted in high yields in mChABC over consecutive days under physiological conditions. Active bacterial ChABC has been secreted from mChABC following the removal of the hydrophobic leader sequence.^36,37^ However, the yield of active ChABC in this form was modest in comparison to that reported here.^37^ Similarly, chondroitinase AC (ChAC) can be endogenously secreted in an active form from mChABC *in vitro* and *in vivo*.^38,39^ Unfortunately, activity characterization of ChAC showed only modest enzyme yield and specific activities on both CS-A and CS-C^40^ in comparison to the values, which we report for mChABC. Moreover, ChAC does not act on DS, which is expressed in the spinal cord following injury,^41^ reducing the enzyme’s effectiveness as a potential SCI treatment. Importantly, we show activity of mChABC is superior to bChABC and other known secreted forms of chondroitinase on all sulfates (CS-A, DS, and CS-C), which predominate in the normal and injured CNS.^42^ This would suggest that the specific modifications made to mChABC cDNA facilitate optimal enzyme secretion and activity within physiological *in vitro* and *in vivo* conditions.

We demonstrate that mChABC operates optimally under the same physiological temperature and pH profile as bChABC.^4,6,33−35^ Moreover, the kinetic profile of mChABC activity shows that the enzymes substrate binding affinity has been reduced, but the catalytic activity of the enzyme has increased as compared to bChABC. This ensures that the catalytic efficiency exhibited by mChABC is similar to the commercial enzyme.^4,6,33−35,43^ However, we uniquely demonstrate that the activity of mChABC significantly exceeded that of the commercial bChABC under optimal physiological conditions due to increased thermostability. mChABC is stable *in vitro* over 3 day at 37 °C, far greater than 24 h achieved by bChABC.^18^ The modifications made to mChABC appear to achieve similar levels of thermostabilization as yielded by those of trehalose.^44^ These data may explain why the modified enzyme has functioned better than the bacterial alternative *in vivo*(31,32,45) and the increase in cellular integration and neurite outgrowth caused by mChABC *in vitro* and *in vivo* when compared to bChABC acting under the same conditions.^29^ The increased thermostability exhibited by mChABC further promotes the use of this second-generation enzyme over the commercially available bChABC.

## Conclusions

mChABC can be reliably and robustly produced from mammalian cell lines. This modified enzyme operates with the same efficiency, optimally under the same physiological conditions, and upon identical substrates as the commercial bacterial enzyme. However, the modified mChABC has increased thermostability at physiologically relevant temperatures, increasing the enzyme functional output compared to the widely used bChABC. These findings support the development of mChABC as a treatment for SCI and other pathological diseases as well as extending our knowledge concerning the expression of prokaryotic genes in eukaryotes.

## Experimental Section

Institutional ethical approval was not required for these experiments, and the study was not preregistered. The recombinant form of bChABC used in this study (mChABC) was based on clone Y133 with mutations at *N*-glycosylation sites Asn 675,282, 345, 515 (S-A), and 715. Please see the study by Muir et al. for further details regarding the modifications made.^28,30^

### Cell Culture Reagents

DMEM with and without phenol red and fetal calf serum (FCS) were purchased from Thermo Fisher Scientific. Penicillin/streptomycin/fungizone (PSF; 2%), trypsin, and poly-d-lysine were purchased from Sigma, while ITS+ (Insulin-transferrin-sodium selerite with bovine serum albumin and linoleic acid; 1:100) was from BD Bioscience. Bacterial chondroitinase ABC (Sigma) was used in a buffer containing 50 mM Tris base and 50 mM sodium acetate (pH 8.0; Sigma). Cell counts were conducted using a Countess automated cell counter (Thermo Fisher Scientific).

### Culture and Transfection of HEK293 Cells

Human embryonic kidney (HEK293) cells were procured from frozen stock populations, thawed, and grown in supplemented DMEM [DMEM with 10% FCS, 2% penicillin/streptomycin/amphotericin, bovine pituitary extract (Sigma, 10 μg/mL)] at 37 °C with 7% CO_2_ and passaged at a ratio of 1:10 every 48 h (or when 70% confluent) using 0.1% trypsin to avoid senescence and increased cell numbers. Stock populations were validated from the producer prior to freezing and underwent no more than 15 passages before being replaced with new stock population. Flasks of cells were assigned to experimental groups using simple randomization, and the experimenter was blind to the treatment groups at all stages of the experiment and analysis.

HEK293 cells were transfected with a Nucleofector (Lonza; program A-23) using Cell Line Nucleofector Kit V (Lonza). Briefly, 2 × 10^6^ cells were trypsinized and resuspended in solution with 5 μg of *p*-mChABC-mCherry, *p*-mChABC, or *p*-mCherry DNA. Following transfection, cells were incubated at 37 °C with 7% CO_2_ and expression of the desired mChABC protein was assessed through immunohistochemistry and the CPC turbidity assay. The rate of cell division and transfection efficiency were determined though immunohistochemistry. The total number of cells was determined through Hoechst-33342 (1:10,000; Sigma) staining, while counter staining with Ki67 (1:200; AbCam) following cellular fixation in 4% paraformaldehyde (Sigma) using previously defined methodology.^29^ Cells were analyzed under fluorescent microscopy (Leica6000).

For the extraction of secreted mChABC, cells were washed and the medium was replaced with DMEM (without phenol red) supplemented with sodium pyruvate (1:1000) and ITS+ (1:100). The medium was collected over the following 24 h with each sample centrifuged to remove cellular debris and EDTA-free protease inhibitor cocktail (Roche) added to ensure any target proteins were not cleaved by endogenous cellular proteases. The remaining supernatant was concentrated (50 K centricon; Millipore) and stored at −20 °C until required for experimentation.

### CPC Turbidity Assay

Using a modified and validated form of the CPC assay,^25,29^ 5 μL of samples from transfected cells was incubated with 50 μL of chondroitin sulfate A (CS-A; 20 μg; Sigma) at 37 °C for 30 min and then denatured at 95 °C. 20 μL of each sample was incubated with an equal volume of the CPC reagent [1:1 of 0.2% (w/v) CPC and 133 mM magnesium chloride; Fluka]. Absorbance was measured at 405 nm using a μQuant Microplate Spectrophotometer (Biotek Instruments), and data were adjusted for baseline based on the negative control. Addition of DNase (Thermo Fisher Scientific) failed to alter optical density data. Using a calibration curve generated with known quantities of bChABC (Sigma), the quantity of the active enzyme in each sample was determined based on the measured absorbance. Calculations were assessed through three separate experiments where, in each, five known concentrations of the enzyme were accurately calculated using this methodology.^29^

### Characterization of ChABC Activity

Enzyme activity was defined as the amount of the product formed by an enzyme per milligram of the total protein (μmol min^–1^ mg^–1^). Solutions were made in 50 mM Tris–HCl (p*K*_a_ = 8.06) and 50 mM NaAc buffer. This buffer was utilized in all experiments as per manufacturers’ guidance to facilitate the activity of bChABC (Sigma Aldrich), as it could accommodate a large pH range.^6,33^ Samples of mChABC from transfected HEK293 cells (collected over 24 h) or bChABC (each 2 μL at 0.5 μg/μL as determined through the CPC turbidity assay) were mixed in an excess of the GAG substrate (400 μL at 1 mg/mL) in a quartz cuvette. The cuvette was immediately placed in a temperature-controlled spectrophotometer (Lambda 35 UV/VS spectrometer, Perkin Elmer), and change in absorbance was monitored every 0.2 ms for 5 min at 232 nm, a wavelength corresponding to the absorbance of disaccharides produced from enzymatic activity.^6,33,43^ Enzyme samples were kept on ice prior to the commencement of the assay, while the substrate was prewarmed to the temperature at which the experiment was conducted. A 2 μL sample of buffer solution was assessed as a negative control. All commercial bChABC enzymes were incubated in DMEM (without phenol red) for 24 h at 37 °C prior to analysis, so as to match the environmental conditions of the mChABC. Quantities of all active enzymes used were determined using the CPC turbidity assay and protein concentration assessed by absorbance at 280 nm on the spectrophotometer.

To acquire enzyme activity, absorbance was corrected for background based on the negative control. The rate of absorbance change was calculated by linear regression (Prism). This was converted to concentration and enzyme activity assessed through Beer-Lamberts Law [rate of absorbance change = molar absorption coefficient (ε) × concentration × cuvette path length], assuming that the chemical equilibrium remained constant. ε of ChABC is 3800 M^–1^ cm^–1^,^6,33,43^ and experiments were carried out at a 1 cm path length. The protocol described was validated using Seikagaku bChABC (not used in further experiments, as it is no longer commercially available), and the values obtained were consistent with those reported in the literature.^4,6,33−35^

To characterize mChABC activity, the enzyme was assessed under of a variety of conditions to determine substrate specificity, optimal pH and temperature, and enzyme kinetics.

#### Substrate Specificity

Determined using four GAG substrates: CS-A from bovine trachea (Sigma), dermatan sulfate (DS or CS-B) from porcine intestinal mucosa (Sigma), CS-C from shark cartilage (Sigma), and hyaluronan (HA; of medium molecular weight—250 kDa) from *Streptococcus pyogenes* (R&D).

#### Optimal pH, Temperature, and Thermostability

The influence of pH was investigated on CS-A and HA at 37 °C in five pHs (pH 5.0, 6.0, 7.0, 8.0, and 9.0). Optimal temperature was assessed by varying the temperature at which the reaction occurred on CS-A at six intervals (25, 30, 37, 40, 45, and 50 °C). To determine thermostability, the enzyme activity on CS-A at 37 °C was measured after enzyme preincubation for 5, 15, and 30 min at 37 and 50 °C.

#### Enzyme Kinetics

CS-A was dissolved at eight concentrations (0.0, 0.01, 0.02, 0.05, 0.1, 0.3, 0.5, and 1.0 mg/mL) in reaction buffer at pH 8.0, and initial reaction rates were recorded every 0.2 ms for 5 min at 37 °C. The data were interpreted using linear regression analysis (GraphPad Prism). The initial rate of reaction (ν0) was determined from the value of the slope from the plot of product formation as a function of time. This value was corrected for background based on the negative control. By analyzing the rate of reaction change at each substrate concentration using Michaelis–Menten equations (with Briggs–Haldane alterations; GraphPad Prism), the components of enzyme kinetics were calculated. This includes the maximal/limiting velocity of an enzyme, as substrate concentration gets large (*V*_max_), the concentration of the substrate leading to half-maximal velocity (*K*_m_), and the number of substrate molecules each enzyme site converts to product per unit time (*k*_cat_). The values of *V*_max_ and *K*_m_ were extracted from the Hanes plot generated by monitoring the product formation and using the equation: [S]/ν = *K*_m_/*V*_m_ + [S]/*V*_m_, where *K*_m_ represents the substrate concentration at half saturation and [S] the substrate concentration. *k*_cat_ was calculated using the equation *V*_max_ = *k*_cat_ × [E], where [E] represents total enzyme concentration. From these values, the catalytic efficiency (*k*_cat_/*K*_m_) was determined.

### Statistics

Power analysis using G*Power was conducted prior to all experiments to ensure that sample sizes used were sufficient to yield reliable data based on known standard deviations to determine the expected effect size, level of acceptable significance, and type 1 error threshold (α) ≤ 0.05 and power (1 – β) ≥ 0.90. All experiments were analyzed blind to the experimental condition, and none were excluded based on the outcome. A minimum of three repeats were conducted for each experiment with separate samples being collected from independent cell culture preparations per condition. The parameters were compared between the control and the test group using either the one- or two-way analysis of variance (ANOVA) with post-hoc Turkey or Bonferroni (defined in the text; GraphPad Prism v9). Divergences were considered significant if *P* < 0.05. Significance values represented as * = *P* < 0.05, ** = *P* < 0.01, *** = *P* < 0.001, and **** = *P* < 0.0001. Data show means ± SD.

## Supporting Information Available

Figures showing expression and secretion of active mChABC-mCherry from HEK293 cells and the chemical structure of chondroitin sulfate A (CS-A), chondroitin sulfate B (dermantan sulfate, DS), chondroitin sulfate C (CS-C), and hyaluronan (hyaluronic acid, HA) (PDF)

## Accession Codes

Bacterial chondroitinase ABC—UniProtKB P59807.

## Notes

Correspondence and requests for the material should be addressed to P.M.W., philippa.warren@kcl.ac.uk. The data sets generated and/or analyzed during the current study are available from the corresponding author on reasonable request.
