# Supplementary material for: Substrate Specificity and Biochemical Characteristics of an Engineered Mammalian Chondroitinase ABC
Source: ACS Omega. 2021 Apr 19;6(17):11223–30. doi: 10.1021/acsomega.0c06262 (PMC8153898; doi:10.1021/acsomega.0c06262)
Supplement: Supplementary file 1 — ao0c06262_si_001.pdf [file ao0c06262_si_001.pdf]

Substrate specificity and biochemical characteristics of an engineered mammalian chondroitinase ABC

AUTHOR NAMES: Philippa M. Warren[a,b,c], James W. Fawcett[a,d#], Jessica C. F. Kwok[a,d,e#]*

** denotes corresponding author; # co-senior author*

AUTHOR AFFILIATION:

[a] Department of Clinical Neurosciences, John van Geest Centre for Brain Repair, University of Cambridge, Cambridge CB2 0PY, United Kingdom

[b] Wolfson Centre for Age Related Diseases, Institute of Psychiatry, Psychology and Neuroscience, King's College London, Guy's Campus, London Bridge, London, United Kingdom

[c] Department of Physiology, Development and Neuroscience, University of Cambridge, Cambridge CB2 0PY, United Kingdom

[d] *Centre for Reconstructive Neuroscience, Institute of Experimental Medicine, Czech Academy of Sciences, Videnska 1083, 14220 Prague 4, Czech Republic*

[e] *School of Biomedical Sciences, Faculty of Biological Sciences, University of Leeds, Leeds LS2 9JT, United Kingdom*

SUPPORTING INFORMATION:

Supplementary Figures: 2

SUPPORTING INFORMATION:

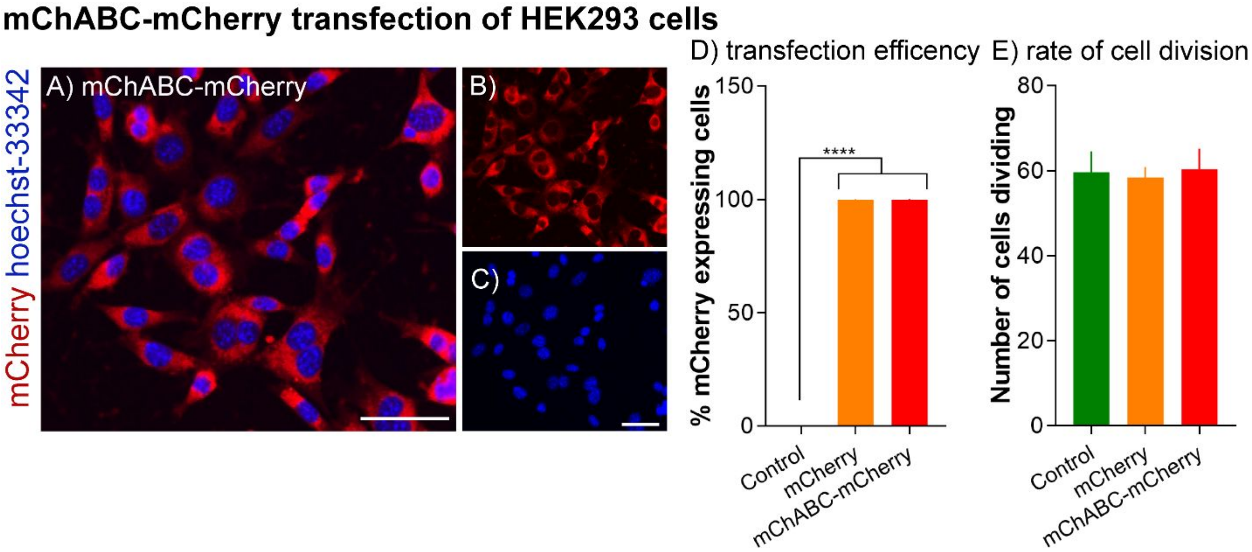

Figure S1: Expression and secretion of active mChABC-mCherry from HEK293 cells. A-C) mChABC-mCherry is readily transfected into HEK293 cells. A) HEK293 cells were either naïve (control), or transfected with mCherry or mChABC-mCherry. Image shows B) mCherry fluorescence (red) and C) Hoechst-33342 (blue) staining. Images show individual channels and a merge. Scale bar = 40 µm. D) Transfection efficiency for mCherry and mChABC-mCherry are ~100% (N=6 independent cell batches assessed per group) and not divergent from each other (P=0.825) although both are divergent from control ($F(2,15)=0.618$, $P<0.0001$; ANOVA with post-hoc Turkey). E) Using anti-Ki67

staining, percentages of HEK cell division was not altered by transfection (N=8 independent cell batches assessed per group; F(2,21)=0.434, P=0.654; ANOVA with post hoc Turkey). For panels D and E, data shows means ± SD.

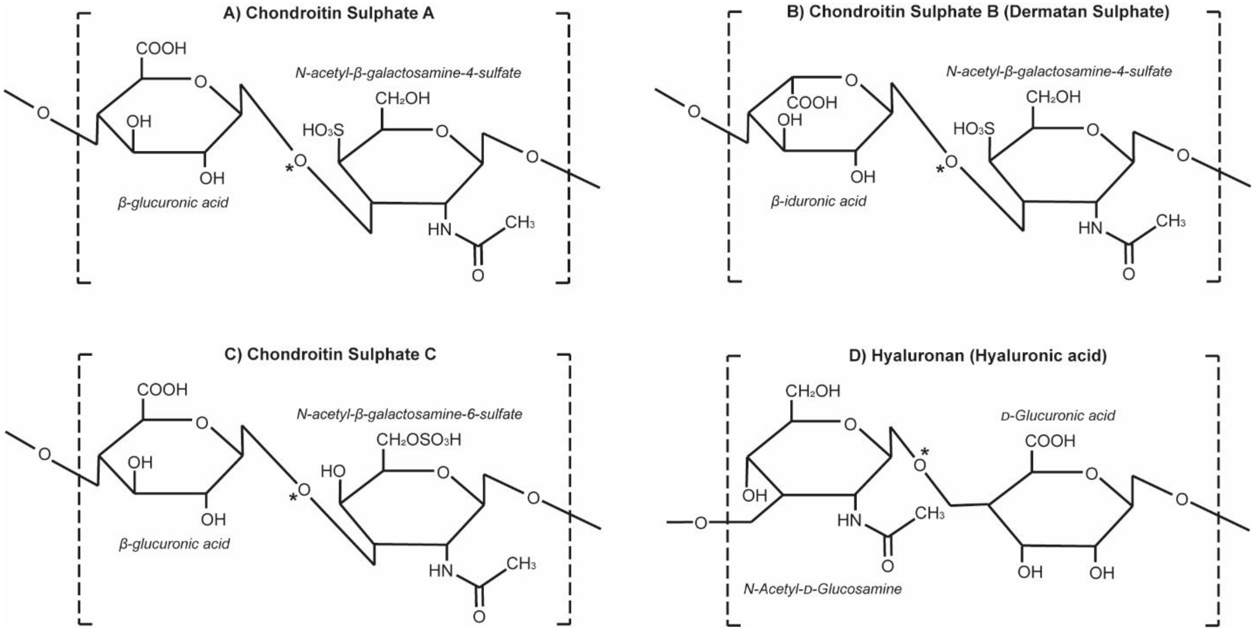

Figure S2: Chemical structure of chondroitin sulphate A (CS-A), chondroitin sulphate B (dermantan sulphate, DS), chondroitin sulphate C (CS-C), and hyaluronan (hyaluronic acid, HA). A-C) Chondroitin sulphate and dermatan sulfate are composed of disaccharides of N-acetylgalactosamine and an uronic acid joined by β(1→4) or β(1→3) linkages, respectively. A) CS-A contains glucuronic acid and is 4 O-sulfated (the

alternating co-polymer β-glucuronic acid-(1-3)-N-acetyl-β-galactosamine-4-sulfate). B) DS contains N-acetylgalactosamine, but the uronic acid present is L-iduronic acid (the alternating co-polymer β-iduronic acid-(1-3)-N-acetyl-β-galactosamine-4-sulfate). C) CS-A contains glucuronic acid and is 6 O-sulfated (the alternating co-polymer β-glucuronic acid-(1-3)-N-acetyl-β-galactosamine-6-sulfate). D) HA is comprised of alternating β-D-(1→3) glucuronic acid and β-D-(1→4)-N-acetylglucosamine residues. For all panels: * represents the point at which chondroitinase ABC would cut break the disaccharides/residuals apart.
